# Patients’ perceptions on reasons for self-referring to the emergency department shortly before a cancer diagnosis: a qualitative study

**DOI:** 10.3399/BJGP.2024.0355

**Published:** 2025-08-12

**Authors:** Xavier Bosch, Elisabet Montori-Palacin, Pedro Moreno, Ana-Maria Guio, Alfonso López-Soto

**Affiliations:** 1 Department of Internal Medicine, Hospital Clínic de Barcelona, August Pi i Sunyer Biomedical Research Institute (IDIBAPS), and Clínic Foundation for Biomedical Research (FCRB), Barcelona, Spain

**Keywords:** cancer diagnosis, decision making, emergency presentation, patient perspectives, primary health care, qualitative research

## Abstract

**Background:**

Some patients are diagnosed with cancer following self-referral to the emergency department (ED), even after consulting in primary care; however, the rationale and factors involved in the decision to self-refer are largely unknown.

**Aim:**

To explore patients’ perceptions on reasons for emergency self-referral shortly before a cancer diagnosis.

**Design & setting:**

Qualitative interview study at a high-volume public institution in Barcelona.

**Method:**

Semi-structured interviews were conducted with two patient groups: patients who self-referred as emergencies and never consulted primary care (non-consulters), and patients who self-referred despite consulting primary care (consulters). Data were analysed by two independent coders — an emergency doctor and a primary care physician — using a codebook approach to thematic analysis.

**Results:**

Fifteen non-consulters and 17 consulters were interviewed. Non-consulters were more likely to belong to disadvantaged and minoritised ethnic communities. Reasons why participants self-referred to the ED were categorised under four themes: urgency/distress, advantages of the ED, quality care, and access to primary care. There was little variation between patient groups in their experiences and perceptions regarding pain intensity and related distress, and EDs’ advantages in terms of accessibility and convenience. Cancer fear, uncertainty about symptoms, and frustration in accessing primary care due to language barriers were unique among non-consulters, leading to help-seeking delays. Patients’ perception of the ED as a facility that provides high-quality care and is able to meet all medical needs emerged as a distinct theme among consulters.

**Conclusion:**

Healthcare organisations and public health services bear the responsibility to promote patient education and improve communication regarding the specific roles and purposes of primary care and the ED. Increasing awareness and developing community-based programmes that target cancer fear and fatalism may encourage early presentation to primary care, especially among underrepresented and minoritised ethnic groups.Delete

## How this fits in

There is a gap in current understanding about why some patients who are diagnosed with cancer following emergency presentation make the decision to self-refer directly to the emergency department (ED), even after consulting primary care. This qualitative study ascertained participants’ key drivers for self-referral. The results underscore the responsibility of healthcare organisations and public health agencies to promote patient education and improve communication about the distinct roles of primary care and the ED, and highlight possible areas for further research and service redesign. Targeted efforts are needed to encourage early consultations in primary care among people with limited resources and from minoritised ethnic groups.

## Introduction

Although the consequences of cancer diagnosis through emergency presentation are well documented and represent a public health concern,^1^ there remain unaddressed gaps in the current evidence: for example, the extent to which primary care physicians (PCPs) contribute to emergency presentations is uncertain.^2^ UK research indicated that approximately a third of patients who presented at the emergency department (ED) were referred there by PCPs, a third consulted primary care services for their symptoms but were not referred as emergencies, and a third never consulted primary care.^3,4^ Despite the frequency of emergency presentations in patients not referred from primary care, evidence on the topic is limited — yet cancer diagnosis through this ‘subroute’ may negatively impact patient prognosis.^5,6^ An analysis of 1802 patients in Scotland diagnosed with cancer through cancer referral pathways revealed that failure to consult primary care beforehand was a strong independent predictor of diagnosis via emergency presentation.^5^

Because of the poor prognosis associated with emergency presentations of patients diagnosed with cancer shortly afterwards, decreasing their frequency is a priority for health systems.^7,8^ Some approaches have yielded promising results: as an example, England is experiencing a marked decline in emergency presentations as PCPs rely more on the 2-week-wait referral system for suspected cancer.^4^ Answering the question of how many emergency presentations in patients not referred from primary care could have been avoided, requires an understanding of why they decided to present themselves as emergencies.^8^

In 2014, Lyratzopoulos *et al* argued that the first presentation as an emergency could be the result of: highly aggressive tumours giving no, or minimal, symptoms before onset of a life-threatening emergency; practical barriers preventing first presentation to primary care; and patient factors, such as cancer symptom knowledge, beliefs, and attitudes or preferences.^8^ Patients’ experiences of cancer diagnosis through emergency presentation were examined in 2015 by Black *et al* using a thematic analysis approach; they reported that most patients experienced repeated cycles of help-seeking, appraisal, and reappraisal for concerning symptoms before emergency presentation, which resulted in diagnostic delays.^9^

Based on evidence showing that patients’ timely recognition of cancer symptoms is associated with earlier presentation and diagnosis, and better outcomes, a range of qualitative studies have investigated symptom appraisal and help-seeking behaviours among patients with cancer.^10,11^ Help-seeking triggers usually included pain, symptom persistence, and advice from family or friends, whereas not recognising symptom seriousness, fear, worry about wasting a physician’s time, and a lack of confidence in the healthcare system often acted as barriers.^12–14^

Against this background, the study presented here aimed to explore patients’ perceptions of reasons for self-referring to the ED, which occurred shortly before they were diagnosed with cancer.

## Method

### Study design and setting

A qualitive study using semi-structured interviews with patients diagnosed with cancer following emergency self-referral was conducted in Hospital Clínic de Barcelona. With an estimated catchment area of 600 000 people, this high-volume public institution offers specialised care to adults referred from 14 associated primary care centres and others visiting the ED.

The Spanish health system provides free access to healthcare services for 99% of the population.^15^ Although Spanish people have access to PCPs in and out of hours, they also have the option to go directly to the ED without first consulting in primary care. Most Spaniards have health insurance, predominantly via public cover, and ~25% use a combination of public and private cover.^15^^,^^16^ Shortcomings associated with the primary care system include, among others, a lack of safety-netting schemes for suspected cancer and restricted access to direct testing. A survey by the Centre for Sociological Research found that the average wait for a primary care appointment in October 2023 was 9.5 days, the longest since records began; although 81% of interviewees held positive views about primary care professionals, 11% reported that they were unable to consult primary care services for a ‘serious health problem’.^17^

### Recruitment and sampling

Participants were purposefully sampled from 316 adults (aged ≥25 years), who had been diagnosed with cancer shortly after self-referring to the ED between September 2021 and December 2023. Potentially eligible patients were identified by two authors who examinined the digital medical records (DMRs) of the hospital and primary care centres. Based on previous utilisation of primary care services, two patient groups could be differentiated:

- those who self-referred and had not consulted primary care (non-consulters); and
- those who self-referred despite consulting primary care (consulters).

By accessing primary care DMRs, the authors were able to assess the number of times each consulter visited a PCP for cancer symptoms in the 12 months before diagnosis. Because of the variety of ethnic groups and language barriers, patients lacking proficiency in Spanish or Catalan were excluded. Patients who were terminally ill or deceased, those unable to communicate verbally, and those who had incidental cancer diagnoses or diagnoses without histopathological confirmation were also excluded. An incidental diagnosis was defined as a cancer diagnosis in the absence of symptoms or due to unrelated symptoms.

Patients who were eligible to participate were first contacted by telephone or email to discuss details of the study. Those who met the inclusion criteria and consented to participate were sent an information pack by email or by post. The pack contained an informed consent form, along with a self-report questionnaire (Supplementary Box S1), which collected quantitative information on:

- sex;
- age at diagnosis;
- marital status;
- living arrangements;
- employment status;
- educational attainment;
- area socioeconomic status — that is, area-based annual income (€/year) provided by the Catalan Health Surveillance System and the Barcelona City Council;^18^
- ethnic group;
- insurance cover;
- smoking habit;
- alcohol consumption;
- comorbidity burden, as measured using the Charlson Comorbidity Index;^19^ and
- symptom burden, as measured using the Edmonton Symptom Assessment System (ESAS) scale, a reliable tool for evaluating the symptom burden of cancer shortly after diagnosis and at the end of life.^20,21^

Participants were included in the study when they had signed and returned the consent form.

By using a ‘code meaning’ strategy, the researchers ensured that the number of participants included reflected a wide range of data issues and a rich understanding of them, as recommended by Hennink *et al*.^22^ Although it was initially estimated that interviewing 22 participants, at most, would suffice, the diversity and complexity of patients’ narratives and unique characteristics of patient groups made this number inadequate for addressing most of the topics. The researchers, therefore, decided to move forward with 10 additional interviews to secure a thorough representation.

### Researcher roles

The research team comprised five internal medicine specialists and co-authors (including one with competence in qualitative research), two professional transcribers, and two clinicians with coding expertise (including an emergency physician and a PCP). All researchers held professional or academic roles at Hospital Clínic de Barcelona and/or the University of Barcelona. Two internists participated in the recruitment and sampling of participants and three developed a topic guide to lead the interviews, which were conducted by the internist who had expertise in qualitative research. The professional transcribers transcribed the interviews, and the emergency physician and PCP built a codebook, and coded and analysed the data.

### Reflexivity

The researchers engaged in a reflexivity process to ensure rigour and minimise researcher bias. Measures were implemented to account for the impact their professional and sociocultural background, as well as personal assumptions, might have on data collection from participant interactions, data analysis, and interpretation of the findings. Although each physician involved in the research had experience in the diagnosis and management of patients with cancer, none had had previous interactions with the study participants.

### Data collection

Interviews were conducted between January and March 2024 via video-conferencing software (Zoom) or the telephone, depending on the participant’s preference, and were recorded. Using the topic guide (Supplementary Box S2) as a reference tool during the interview process, participants were encouraged to elaborate on their rationale for self-referring to the ED and to share insights on how presenting symptoms influenced their decision. They were also asked to describe how their perception and previous knowledge of primary care and emergency services impacted their decision making, and whether their choices were motivated by pre-existing preferences for using emergency services. Interview duration ranged from 37 minutes to 53 minutes.

### Data analysis

Interview recordings were transcribed verbatim, anonymised, and imported into NVivo (version 12) software for data management. Data were analysed using a codebook approach to thematic analysis, which uses the dualistic technique of deductive and inductive thematic analysis. A codebook provides a thorough and detailed account of the hierarchical relationships that exist among codes or themes, including descriptions of each code or theme, illustrative examples, and other relevant details.^23–25^ The analysis process started with deductive orientation, whereby researchers approached the data with existing evidence, theory/theories, and concepts. In addition to Lyratzopoulos *et al*’s proposed taxonomy of mechanisms contributing to emergency presentations and emergency self-referrals,^8^ and physicians’ prior clinical expertise, data collection was informed by qualitative studies on patients’ experiences and perceptions of cancer symptoms and help-seeking behaviours,^10,12,13,26–28^ and studies on patients’ perspectives regarding motives for self-referring to the ED for benign conditions.^29–32^ Accordingly, an initial codebook with a predefined set of codes was constructed.

After reading and re-reading individual transcripts, the emergency physician and PCP, working independently as coders, assigned the predefined codes to raw-data segments associated with the issues raised by participants. To ensure the validity of the research, all transcripts were double-coded by the two independent researchers.

Although initially deductive, coding evolved into a broader inductive approach in order to capture patterns of shared meaning (that is, themes) within the datasets. Candidate themes needed to reflect the diversity of meanings around the primary organising concept, and demonstrate consistency across the whole dataset and the research question. The subsequent phase of defining themes allowed for those that contained complex meanings to be split, thin themes to be merged or discarded, and clear subthemes to be developed. Once defined and named, themes and subthemes were incorporated into the codebook, which was arranged hierarchically, with broad themes leading to more focused ones.

The research team held regular meetings to resolve coding discrepancies and facilitate reflexivity. The final codebook was agreed by all authors. The findings are reported according to the Standards for Reporting Qualitative Research.

## Results

Thirty-two patients were interviewed: 15 non-consulters and 17 consulters. In the 12 months before diagnosis, six (35%) consulters had attended primary care two or three times, seven (41%) had attended between four and six times, and three (18%) did so seven or more times.

Participant characteristics are detailed in Supplementary Table S1. Non-consulters had a mean age of 63 years and 47% were women; the mean age of consulters was 68 years and 41% were women. Although consulters had a greater comorbidity burden than non-consulters, the latter were more likely to live alone, be unemployed, belong to non-White ethnicities, and have lower income and education levels. Cancer types included lung, pancreatic, colorectal, upper gastrointestinal (gastric and oesophageal), ovarian, breast, and prostate cancer.

Patients from both groups — but more notably consulters — reported a high prevalence of moderate-to-severe scores for pain on the ESAS scale (Supplementary Table S2). The symptom profile of pancreatic, gastric, and ovarian cancer was mainly characterised by non-specific or vague symptoms and only one or two localising (that is, ‘red-flag’) symptoms. Consulters were more likely than non-consulters to present with non-specific symptoms and be diagnosed with pancreatic and ovarian cancer, whereas non-consulters were more likely to have breast cancer, characterised by a narrow symptom signature (that is, breast lump/nipple changes) (data not shown).

Four main themes were identified as reasons for self-referring to the ED:

- urgency/distress;
- advantages of the ED;
- quality care; and
- access to primary care.

**Figure 1.**
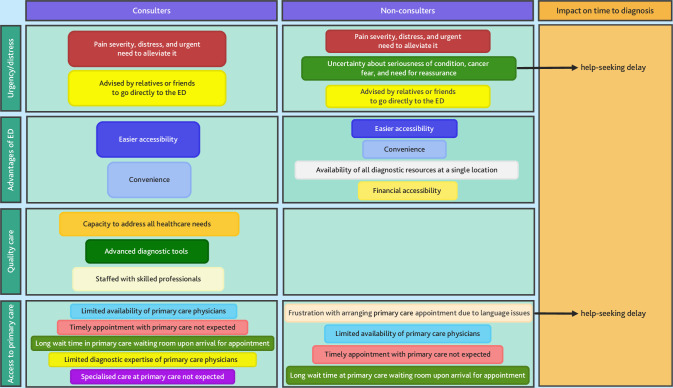
Themes and subthemes relating to emergency self-referral in patient groups with and without previous consultations in primary care. ED = emergency department

Preferences for emergency versus primary care services had a marginal role and were not considered a relevant theme. Pain, either alone or concurring with anxiety and wellbeing, influenced self-referral decisions in most patients (see ESAS scores for these symptoms in Supplementary Table S2). The different themes and related subthemes of both patient groups are illustrated below (Figure 1).

### Urgency/distress

Many patients presented to the ED because of the perceived severity and urgency of presenting symptoms. Most were specifically concerned about the level of pain they were experiencing, with a need for urgent care to alleviate it and the distress it was causing. Pain intensity and associated distress had a significant impact on decision making in both groups, particularly in consulters, both in terms of intensity and duration (see scores for these symptoms on ESAS scale in Supplementary Table S2). One consulter stated:

*‘The pain was unbearable. I’ve never had anything that intense, so I had no choice but to go to hospital*.’ (Consulter [C] 6, male [M], 60 years)

Another consulter reported experiencing severe back pain for several weeks, which his PCP had attributed to a former disc herniation and for which ibuprofen, tramadol, and pregabalin had been recommended:

*‘I told my doctor about my worsening back pain multiple times, but she believed it was just from my past slipped disc*.’ (C4, M, 67 years)

Uncertainty about the seriousness of presenting symptoms and a need for fears to be allayed by seeking reassurance were also key drivers, especially among non-consulters:

*‘I didn’t know what was wrong with me, but I was getting really scared, so I went there* [the ED] *to calm down*.’ (Non-consulter [NC] 3, female [F], 59 years)

One patient expressed concern about the potential for melanoma in a pigmented skin lesion. However, because of a fear of being told that she had cancer by her PCP, she had delayed help seeking for a year, by which time the lesion had grown considerably and additional symptoms had emerged:

*‘I noticed this mole on my leg that was getting bigger and I got worried it might be cancer. But I was too freaked to go to the* [primary care] *doctor, so I just left it alone for a year until I ended up in the ED and they diagnosed me with melanoma that had spread to my bones*.’ (NC5, F, 58 years)

Another patient decided to wait before seeking elective care, hoping her symptoms would subside without intervention, but the condition became an emergency:

*‘I was having some stomach pain for around two months and I thought it was just gas. I waited it out but it got too intense, so I had to rush there* [to the ED].’ (NC9, F, 75 years)

One patient from each group, worried about the intensity and underlying nature of their condition, consulted with family members; both were advised to go directly to the ED, rather than seeing their PCP:

*‘My sister said I had to get help right now, so I quickly went to the ED*.’ (C1, F, 65 years)

*‘I talked to my wife about how much I was hurting. She could see it was serious, so we decided to go straight* [to the ED].’ (NC1, M, 62 years)

### Advantages of the ED

An important trigger for emergency self-referrals among non-consulters was the perception that, at the ED, all diagnostic testing that might be required was readily available at a single location, unlike in primary care, where multiple appointments were likely to be needed:

*‘I had to get some testing done quickly, and they* [at the ED] *were all prepared. Seeing your regular doctor is just for routine check-ups, nothing major going on*.’ (NC14, M, 73 years)

Accessibility and convenience influenced decision making in both groups. Some patients perceived the ED as providing unrestricted care:

*‘You can always count on the ED; there are no restrictions when you need it*.’ (NC2, F, 64 years)

The ED was also seen as a place where it was possible to receive uninterrupted medical care, and was considered to be more easily accessible than primary care. In some patients, seeking help from the ED was a more convenient way to be able to resume daily routines:

*‘I gotta heal quickly, since I have to be back on the job tomorrow*.’ (C3, F, 77 years)

A patient lacking health insurance, who was not registered with a primary care centre, stated that he decided to self-refer to the ED because he knew he could access care there:

*‘No insurance or regular doctor for me, but I can still go to the ED if I have to*.’ (NC4, M, 67 years)

### Quality care

Several patients reported that their decision to seek emergency care over primary care was influenced by their perception of the ED as a facility offering high-quality care and one that would be able to meet all of their healthcare needs (for example, pain control). Under this theme, patients emphasised the 'quality of care' of emergency services rather than their 'advantages' in terms of accessibility, availability of diagnostic tests, or convenience. As one participant noted:

*‘When you’re in pain and need to feel better, the ED is where it’s at. And they’ll give you specialised care for your specific illness*.’ (C17, M, 70 years)

Some consulters viewed the ED as a facility with advanced diagnostic tools and skilled professionals who could diagnose and treat their condition effectively:

*‘Those doctors* [in the ED] *are quick and well trained. They know exactly how to diagnose and fix the problem*.’ (C10, M, 81 years)

*‘They* [in the ED] *have all kinds of advanced devices for figuring out what’s wrong*.’ (C5, F, 55 years)

Two consulters’ decision-making processes were shaped by their perception that primary care did not offer the specialised care found in the ED or that PCPs lacked the necessary expertise to diagnose serious conditions:

*‘The general* [primary care] *centre doesn't offer the same specialised care as the ED*.’ (C8, M, 72 years)

*‘The* [primary care] *doctor's not the most skilled at catching serious medical conditions.*’ (C14, M, 58 years)

### Access to primary care

Patients’ choices to seek help from the ED were partly determined by perceived and practical barriers to accessing primary care. Emergency services were often accessed due to a generally held belief — sometimes based on previous experiences or acquired from relatives and friends — that PCPs were not readily available at the time of feeling unwell or that waiting times at a PCP’s office on arrival for an appointment were excessive:

*‘She* [PCP] *was working that day but she’s only there part time. She’s the best, so I tried to get an appointment five hours before she was supposed to start. But the lady at the desk was like, “Sorry, she’s completely booked. No room for you*.’ (C12, F, 69 years)

One consulter, who was experiencing pain related to his cancer and had already attended primary care three times, believed that a further primary care appointment would not be available within an acceptable time period and decided to self-refer to the ED when his pain became intolerable:

*‘I kept going to the doctor for my abdominal pain but, every time I tried to make an appointment, it took an eternity … then it got so bad that I had to go to hospital right away.’* (C9, M, 73 years)

A non-consulter, who had previously visited his PCP for an unrelated issue, stated:

‘*It’s gonna be tough to see him while you’re sick; you’ll have to wait ages to get an appointment*.’ (NC10, M, 55 years)

Two non-consulters, who were from Asia and for whom Spanish was a secondary language, faced challenges in effectively communicating their appointment requirements via telephone, resulting in feelings of frustration. One of them voiced frustration at not being able to understand the instructions given for having a primary care appointment:

*‘After losing weight and having some abdominal discomfort, I got very concerned when my husband and son saw my eyes and skin were yellow. I first tried to do the right thing and contacted the [primary care] centre for an urgent doctor's appointment. But despite I kept saying I really needed to see someone fast, I just couldn't get what the person on the phone was telling me to do. So, I hit up the ED and got hospitalised.’* (NC7, F, 60 years)

## Discussion

### Summary

This research examined patients’ perceptions and experiences of why they decided to self-refer as emergencies before being diagnosed with cancer. Findings suggested there were opportunities to support informed decision making for patients, physicians, and the broader health system. Although a complex interplay of mechanisms influenced patients’ decision making, an urgency to alleviate pain and uncertainty about the nature and seriousness of presenting symptoms, along with a need for reassurance, were key determinants for emergency self-referrals. Patients’ expectations for high-quality care in the ED and its potential to provide better resources, expertise, and accessibility relative to primary care also played a decisive role.

Although themes and subthemes differed between the two patient groups, there was little variation in their experiences and perceptions regarding pain intensity, associated distress, and the ED’s perceived advantages regarding accessibility and convenience. However, the experience of pain and a need for immediate relief appeared to be a more pressing concern for consulters; this was consistent with their higher rates of moderate-to-severe ESAS scores and may have been reflected in the fact that, at diagnosis, they had later-stage cancers. Uncertainty and fear about a possible cancer diagnosis, along with a belief that symptoms would resolve by themselves, were unique among non-consulters, and resulted in help-seeking delays.

Patients’ views about the quality of care received in the ED emerged as a clear and distinct theme among consulters; they viewed the ED as a versatile facility with qualified health professionals and advanced diagnostic tools, which was ready to address a variety of medical needs. Although both groups shared the perception that obtaining a timely primary care appointment was unlikely, their views also differed to some extent: frustration at not being able to arrange a primary care appointment due to limited language skills that restricted the ability to effectively communicate their needs over the phone highlighted a distinct issue for some non-consulters, whereas consulters felt that primary care did not provide the necessary knowledge, medical resources, or specialised services that were readily accessible in the ED.

### Strengths and limitations

A key strength of the study is the fact that two separate patient groups with different sociodemographic and clinical characteristics were analysed; this expands the scope of perspectives in the overall population. In addition, having a PCP and an emergency physician, both skilled in coding, undertaking an independent dual-coding analysis of the transcripts substantiated the reliability and integrity of the findings, and contributed to enhance data quality and reduce potential bias. However, some limitations must be acknowledged. The findings may reflect the organisation of local emergency and primary care services, and may not be transferable to other settings. Thematic analysis was conducted based on Braun and Clarke’s (2006) principles^24^ and — even though different thematic analysis approaches were clearly delineated in subsequent versions of their work — the authors of the study presented here, used a codebook approach that combined a structured coding method (with codebook) with the qualitative principles of reflexive thematic analysis.^33–35^ How patients’ knowledge of cancer symptoms shaped emergency self-referral decisions, and why PCPs decided against referring to the ED patients with whom they had interacted , were not explored. Last, even though we adopted a 'code meaning' strategy and interviewed 32 patients, the establishment of two separate groups, one with 15 and the other with 17 patients, implied that the sample sizes were relatively small, representing a limitation for the research.

### Comparison with existing literature

In their study of patients’ experiences of an emergency cancer diagnosis, Black *et al* described two patients who bypassed primary care after numerous unsuccessful visits and decided to self-refer as emergencies; they also reported that, in some cases, an escalation of symptoms was a key factor leading to cancer diagnosis.^9^ These results align with this study’s findings regarding patients who self-referred when their symptoms— especially pain — intensified, despite repeated consultations. Black *et al* also documented instances when patients were given a benign diagnosis rather than one of cancer, which is similar to the experience of the consulter in the study presented here, who returned to primary care for worsening back pain that was misattributed to a herniated disc.^9^

An analysis of non-consulters' sociodemographic data indicated that they experienced higher unemployment rates, frequently lived alone, and were often from minoritised ethnic and economically challenged populations. Evidence demonstrates that risk of cancer diagnosis via emergency presentation is influenced by socioeconomic and ethnic factors, and that patients with cancer who have a low income and present as emergencies often lack prior primary care consultations; this reflects practical or emotional access barriers.^3,27,36^ Cancer fear and concerns regarding medical findings often create emotional barriers to accessing primary care for patients who have cancer and are from minoritised and low-income populations, thereby impacting timely help seeking and diagnosis.^37^

### Implications for research and practice

Patients' contrasting expectations and perceptions about the services provided by the ED and primary care that were revealed by this study underscore the responsibility of healthcare organisations and public health agencies to promote patient education and improve communication regarding the distinct roles and purposes of each care setting. In England, increased awareness of cancer symptoms and encouraging people to consult primary care for these symptoms have led to the highest rate of early cancer diagnoses ever reported.^38^

Linking non-consulters’ perceptions/experiences with their sociodemographic profile revealed additional implications for primary care. Cancer fear in non-consulters and their beliefs concerning presenting symptoms led to delays in seeking help. Language barriers and low awareness of cancer symptoms increased the challenges, further delaying the diagnostic process. These findings stress the importance of targeted actions to promote early presentation to primary care, including public awareness campaigns and community interventions targeting cancer fear and fatalism in underserved populations. Similarly, culturally appropriate resources and translation services can improve healthcare accessibility for minoritised ethnic groups and so it is important that these are designed and brought into use.

Research has shown that patients’ recognition of the nature of cancer symptoms and PCPs’ timely referrals to specialised care may lead to earlier diagnosis, cancers being at an earlier stage at diagnosis, and improved cancer survival.^27,39,40^ Despite their visiting PCPs repeatedly for severe pain and related anxiety, consulters in the study presented here were not referred as emergencies by PCPs; without such PCP involvement and in facing additional access challenges, these patients may have viewed the ED’s quality of care and accessibility as more valuable in guiding their choices. However, considering that these patients all had cancer, it could be argued that their decision to self-refer and not reconsult in primary care was entirely reasonable. Further qualitative studies could shed light on the impact of patients’ symptom literacy and beliefs on self-referral decisions, as well as PCPs’ rationale for not referring patients with whom they had previously interacted to the ED. The recognised role of PCPs’ gut feelings in guiding patients through cancer diagnostic pathways creates opportunities to investigate physicians’ perspectives on the impact of their use, if any, on their decision-making processes in these particular cases.^41,42^

As service configuration and accessibility play a key role in patients’ decision making, adjusting the acute care delivery system to maximise the advantages of the ED that were identified in this study could improve primary care accessibility and utilisation. Service reconfiguration could involve facilitating direct testing access in primary care, using telephone triage systems, or implementing safety netting. Assessing the potential integration of PCPs in the ED is also an option.^43^ In England, for example, a £100 million funding-backed policy proposal seeks to establish co-located primary care services in every ED, enabling these facilities to prioritise the care of the most critically ill patients.

## Data

Data are available upon reasonable request to the corresponding author (Xavier Bosch). It should be noted that raw data is only available in Spanish.
